# Bone Marrow Mesenchymal Stem Cells and Their Derived Extracellular Vesicles Attenuate Non-Alcoholic Steatohepatitis-Induced Cardiotoxicity via Modulating Cardiac Mechanisms

**DOI:** 10.3390/life12030355

**Published:** 2022-02-28

**Authors:** Marwa O. El-Derany, Sherihan G. AbdelHamid

**Affiliations:** Department of Biochemistry, Faculty of Pharmacy, Ain Shams University, Cairo 11566, Egypt; dr.sherehan@pharma.asu.edu.eg

**Keywords:** extracellular vesicles, BM-MSCs, mitophagy, NASH, cardiotoxicity, miRNAs

## Abstract

Cardiovascular-disease (CVD)-related mortality has been fueled by the upsurge of non-alcoholic steatohepatitis (NASH). Mesenchymal stem cells (MSCs) were extensively studied for their reparative power in ameliorating different CVDs via direct and paracrine effects. Several reports pointed to the importance of bone marrow mesenchymal stem cells (BM-MSCs) as a reliable therapeutic approach for several CVDs. Nevertheless, their therapeutic potential has not yet been investigated in the cardiotoxic state that is induced by NASH. Thus, this study sought to investigate the molecular mechanisms associated with cardiotoxicity that accompany NASH. Besides, we aimed to comparatively study the therapeutic effects of bone-marrow mesenchymal-stem-cell-derived extracellular vesicles (BM-MSCs-EV) and BM-MSCs in a cardiotoxic model that is induced by NASH in rats. Rats were fed with high-fat diet (HFD) for 12 weeks. At the seventh week, BM-MSCs-EV were given a dose of 120 µg/kg i.v., twice a week for six weeks (12 doses per 6 weeks). Another group was treated with BM-MSCs at a dose of 1 × 10^6^ cell i.v., per rat once every 2 weeks for 6 weeks (3 doses per 6 weeks). BM-MSCs-EV demonstrated superior cardioprotective effects through decreasing serum cardiotoxic markers, cardiac hypoxic state (HIF-1) and cardiac inflammation (NF-κB p65, TNF-α, IL-6). This was accompanied by increased vascular endothelial growth factor (VEGF) and improved cardiac histopathological alterations. Both BM-MSCs-EV and BM-MSCs restored the mitochondrial antioxidant state through the upregulation of UCP2 and MnSOD genes. Besides, mitochondrial Parkin-dependent and -independent mitophagies were regained through the upregulation of (Parkin, PINK1, ULK1, BNIP3L, FUNDC1) and (LC3B). These effects were mediated through the regulation of pAKT, PI3K, Hypoxia, VEGF and NF-κB signaling pathways by an array of secreted microRNAs (miRNAs). Our findings unravel the potential ameliorative effects of BM-MSCs-EV as a comparable new avenue for BM-MSCs for modulating cardiotoxicity that is induced by NASH.

## 1. Introduction

Although liver manifestations are the key characteristic features of non-alcoholic fatty liver disease (NAFLD), extrahepatic multisystem complications have also been widely reported [1,2]. NAFLD results from metabolic derangements due to surplus lipid overloads affecting hepatic structure and function. It eventually progresses into non-alcoholic steatohepatitis (NASH), liver fibrosis, cirrhosis and hepatocellular carcinoma (HCC). However, its severity extends beyond the liver, where the majority of deaths are attributable to cardiovascular diseases (CVDs) [3]. Increased prevalence of coronary artery disease and structural myocardial alterations was reported in NAFLD/NASH [4], suggesting that these associated CVDs may be independent of the presence of traditional cardiovascular (CV) risk factors [4,5,6]. 

Diverse pathogenetic assumptions support the notion of cardiohepatic bidirectional interactions [5]. Beyond insulin resistance, which has been the focus of most previous studies, subclinical inflammation and oxidative stress are considered as key contributors to the pathophysiological mechanisms linking NASH with cardiac complications [6]. At the cellular level, mounting evidence has related the impairment in mitochondrial function to all characteristic features of NASH and its associated complications, including CV events [7]. It is noteworthy that about 30–40% of the cardiomyocyte is composed of mitochondria, and mitochondrial status potentially modulates the cardiomyocyte function [8]. Proper mitochondrial function is indispensably required by the heart owing to its continuous high energy demand [9]. Accordingly, CV events are reported with early stages of NASH, being a primarily mitochondrial disease that affects the overall ATP balance and energy state [10]. 

Deteriorated mitochondria are constantly replaced by processes involving mitochondrial biogenesis and autophagy/mitophagy [11]. Mitophagy is tightly regulated to control mitochondrial dynamic formation and remodeling via the removal of inefficient and dysfunctional mitochondria [12]. Thus, mitophagy helps to maintain the biochemical homeostasis of cells and guards against ATP depletion [13]. However, impaired mitophagy has recently been reported in NASH due to the elicited lipotoxic state in the liver. This impairment subsequently aggravates the NLRP3 inflammasome activation, which drives NASH progression [14]. Unsurprisingly, growing evidence has linked the development of CVDs to mitophagy impairment [15], which has been attributed to both Parkin-dependent and -independent mechanisms in different CVDs [15]. 

Interestingly, a mutual interrelation has been reported between metabolic dysregulations, chronic inflammatory states and the development of chronic cellular hypoxic state in NASH [16]. This chronic hypoxic stress, in turn, disrupts the mitochondrial quality control and induces mitochondrial deficits through impairing mitophagy. Besides, it augments the production of pro-inflammatory cytokines, which ultimately reduces cardiomyocyte viability [17]. Hypoxic states stimulate hypoxia inducible factor (HIF), a pivotal oxygen-sensing transcription factor, which in turn transactivates a wide array of target genes. Vascular endothelial growth factor (VEGF) is one of the primary genes regulated by HIF [18]. As such, the induction and control of angiogenesis is coordinately affected by the interplay of different cardiac cellular hypoxic and inflammatory dysregulations [18]. Nevertheless, the complexity surrounding these impaired cardiac mechanisms in the cardiotoxic state that is induced by NASH is currently far from clear.

Mesenchymal stem cells (MSCs) have been extensively studied for their reparative power in ameliorating different CVDs [19]. Among these are bone-marrow-derived MSCs (BM-MSCs), whose potential therapeutic effects in CVDs were theoretically translated from their wide differentiation capacity [20]. Besides, recent studies revealed that MSCs reparative effects could occur through paracrine manner as well [21,22,23,24,25]. Being one of the best known sources of progenitor cells, BM-MSCs are successfully capable of differentiation into cardiomyocyte, and thus, several reports point to their importance as a reliable therapeutic approach for several CVDs [26,27,28]. More interestingly, attention has been directed toward stem cell secretome to mediate multiple protective and reparative effects, such as, but not limited to, neuroprotective effects [29], hepatoprotective effects [30], cardiac repair [31] and pulmonary repair [32,33,34]. Studies highlighted that extracellular vesicles (EVs) secreted by stem cells can improve cardiac regeneration [35,36]. Nanosized EVs contain bioactive substances, including proteins, lipids and nucleic acids, with important functions in diverse intercellular communications [37,38,39,40,41,42]. Novel regenerative medicine approaches for the treatment of CVDs have considered the utilization of the paracrine and mircrine mechanisms of stem cells exerted via EVs. Recently, BM-MSCs-derived EV (BM-MSCs-EV) were shown to have a protective effect against myocardial infarction by promoting autophagy [43,44]. In particular, MSCs-derived microRNAs (miRNAs) were introduced as important regulators of multiple cardiac mechanisms, facilitating a new path for CVD treatment [45]. 

Accordingly, we aimed to investigate the underlying molecular mechanisms of cardiotoxicity induced by NASH in a rat model. Besides, we sought to elucidate the potential modulatory effects of BM-MSCs-EV and BM-MSCs against cardiotoxic events induced by NASH through epigenetic regulation of the key molecular pathways that control several cardiac mechanisms. We aimed to investigate the miRNA paradigm of BM-MSCs that controls PI3K/AKT, hypoxic, VEGF and inflammatory signaling pathways in cardiotoxic state induced by NASH. 

## 2. Materials and Methods

### 2.1. Animals

Sprague Dawley (SD) rats were purchased from Company of El-Nile for the Pharmaceutical and Chemical industries, Egypt. Their average weights were (120–150 g). Open cages were used and adjusted to 12 h light and dark cycles at a temperature of 25 °C at the animal facility of the Faculty of Pharmacy, Ain Shams University, Egypt. Animals were left for acclimatization before starting the experiment for one week. The experimental protocol was approved by the Research Ethics Committee at the Faculty of Pharmacy, Ain Shams University, Egypt (memorandum no ENREC-ASU-2020-66).

### 2.2. Isolation and Characterization of BM-MSCs and BM-MSCs-EV

Sterile syringes were used to aspire bone marrow from SD rats from the tibia and femurs. BM-MSCs were cultured in F12 Dulbecco’s modified Eagle’s medium supplemented with 10% bovine serum (Lonza, Walkersville, MD, USA), 100 U/mL penicillin, and 100 μg/mL streptomycin (Gibco-BRL, Grand Island, NY, USA). Cells were maintained at 37 °C with 5% CO2 in a humidified incubator as previously described [46]. For characterization, BM-MSCs were stained with FITC-conjugated anti-rat cluster of differentiation 105 (CD105) and FITC-conjugated anti-rat CD45 and CD14 (Beckman Coulter, Brea, CA, USA) as previously described [47]. CYTOMICS FC 500 Flow Cytometer (Beckman Coulter, Brea, CA, USA) was used for cell analysis, and CXP Software version 2.2 was used for analysis.

Multipotent differentiation was investigated for BMSCs. Cells were cultured in adipogenic media (StemXVivo^®^, R&D Systems) for 21 days. The media were replaced every 3 days, and last-day cells were fixed with 10% neutral formalin for 1 h at room temperature. Cells were subsequently washed by 60% isopropanol and dried. Finally, filtered oil red (Sigma-Aldrich, Saint Louis, MO, USA) was added on the cells and incubated for 4 h. Then, cells were washed, air dried, and staining was detected under an inverted phase-contrast microscope (Olympus, Feasterville, PA, USA).

Osteogenic differentiation was conducted by culturing BMSCs in osteogenic induction medium (StemXVivo^®^, R&D Systems) for 14 days. The media were replaced every 2 days. Cells were washed and stained by Alizarin red staining (Sigma-Aldrich, Saint Louis, MO, USA). Stained calcium depositions were shown by the inverted phase-contrast microscope (Olympus, Feasterville, PA, USA).

For isolation of EVs, BM-MSCs were maintained in serum-free conditioned media for 48 h. The media were subsequently collected and differentially centrifuged for 10 min at 300× *g*, for 20 min at 2000× *g*, and for 30 min at 10,000× *g*. Then, the medium was centrifuged at 100,000× *g* for 70 min at 4 °C to isolate the EV pellets. Then, pellets were washed by PBS and centrifuged for 70 min at 100,000× *g* at 4 °C. BM-MSCs-derived EV were suspended in PBS and stored at −80 °C. EV characterization was performed by inspecting the vesicular structure by transmission electron microscope (TEM) (JEM-1010; JEOL Ltd., Tokyo, Japan) at an acceleration voltage of 70 kV. Briefly, 1% phosphotungstic acid, a negative stain, was applied on the adherent EV loaded onto carbon-coated 220 mesh copper grids and left to dry at room temperature for 1 h and inspected under the microscope as mentioned previously [48]. In addition, EV surface markers were determined by Western blot as mentioned previously [49]. The membrane was subsequently incubated with specific antibodies for CD63 (MX-49.129.5): sc-5275 Santa Cruz Biotechnology, Inc., Dallas, TX, USA) and CD81 (B-11): sc-166029 Santa Cruz Biotechnology, Inc., Dallas, TX, USA) at 4 °C overnight. The bands were visualized by chemiluminescence.

### 2.3. Labeling of BM-MSCs with PKH26

BM-MSCs were collected and labeled with PKH26 Red Fluorescent Cell Linker kit (Sigma-Aldrich, St Louis, MO, USA) according to the manufacturer’s protocol. Approximately 1 × 10^6^ cells were collected at passage 4 and labeled. Subsequently, labeled cells were intravenously injected at the seventh week of the experiment. Rats were anesthetized and decapitated 24 h after injection, and heart tissues were collected. Fluorescence microscope was used to detect and trace the labeled stem cells.

### 2.4. miRNA Sequencing and Association with Key Targets and Pathways

BM-MSCs extraction of miRNAs was performed using mirVana™ miRNA Isolation Kit (Thermo Fisher Scientific, Austin, TX, USA). Next-generation sequencing (NGS) was performed using TruSeq small RNA Sample Preparation Kit (Illumina, The Netherlands). Sequencing was performed on a HiSeq 2000 (Illumina) paired-end 100 cycle (PE100) run. Reference rat genome was applied. Annotation of the miRNAs was performed using miRBase (v.19), and miRNAs were fed into the Ingenuity Pathway Analysis (IPA)^®^ software to detect target genes and pathways related to our results of miRNAs list. 

### 2.5. Experimental Design

**Figure 1:**
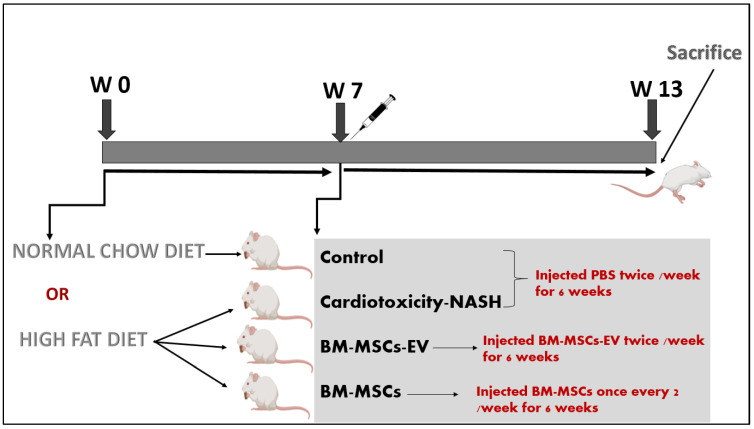
Experimental study design timeline.

The duration of the experimental model was 12 weeks, as shown in Figure 1. The study included 4 equal groups of rats (*n* = 10 rats per group). 

The first group was the control group and was fed with normal chow diet (El-Nasr Company in Abu Zaabal, Egypt) consisting of carbohydrate, protein and fat (53%, 23% and 5%). Rats were given PBS (0.2 mL i.v.) on the seventh week, twice a week for 6 weeks.

The second group was considered as untreated group representing the NASH associated cardiotoxicity. This group was fed by HFD purchased from El-Nasr Company in Abu Zaabal, Egypt consisting of 30% sucrose, 30% lard stearin, 4% palmitic acid, 2% cholesterol and 0.5% cholic acid for 12 weeks. Rats were then given PBS (0.2 mL i.v.) on the seventh week, twice a week for 6 weeks, as previously described [30]. 

The third group was the BM-MSCs-EV-treated group. The rats were fed by HFD, same as the second group. On the seventh week, BM-MSCs-EV at a dose of (120 µg/kg) were given twice a week for 6 weeks (0.2 mL i.v.) (12 doses per 6 weeks). This dose was selected based on the therapeutic dose for NASH, as discussed previously [50,51].

The fourth group was the BM-MSCs-treated group. The rats were fed by HFD. On the seventh week, i.v. injection of BM-MSCs was given to each rat at a dose of 1 × 10^6^ cells, every other week for 6 weeks (three times in 6 weeks). The dose of BM-MSCs was chosen in accordance with previous studies [30,52,53]. 

### 2.6. Histopathological Examination and Scoring

Formalin-fixed liver and heart tissues were stained by hematoxylin and eosin (H&E) (Sigma-Aldrich, St Louis, MO, USA). The histological scoring system for NASH activity score (NAS) was performed blindly to assure the occurrence of NASH.

### 2.7. Assessment of Serum Cardiotoxicity Markers

Serum creatine kinase isoenzyme-MB (CK-MB) and lactate dehydrogenase (LDH) activities were determined using colorimetric kits (Spectrum diagnostics, Cairo, Egypt). Besides, serum cardiac troponin I (c-TnI) level was determined using ELISA kit (Life Diagnostics Inc., West Chester, PA, USA) according to the manufacturer’s protocol. 

### 2.8. Immunohistochemical Analysis

Immunohistochemical analysis was utilized to determine the heart content of vascular endothelial growth factor (VEGF) using ready-to-use primary antibody, monoclonal Anti-VEGF (ab1316-1:200) (Abcam). Moreover, hypoxia-inducible factor-1 (HIF-1) immunohistochemical analysis was carried out using ready-to-use primary antibody, anti HIF-1 alpha Antibody (NB100-123-Novusbio). Immunohistochemical technique was performed. Deparaffinized 5μn thick tissue sections were treated for 20 min by 0.3% H_2_O_2_ (Al Gomhorya, Cairo, Egypt), followed by incubation with primary antibody, and then washed out by PBS (Bio-diagnostic, Cairo, Egypt). Subsequently, slides were incubated for 20 min with secondary antibody HRP Envision kit (DAKO, Ely, UK), then washed and incubated for 15 min with diaminobenzidine. Finally, slides were washed out by PBS, then counter-stained with hematoxylin, dehydrated and cleared in xylene, then covered for subsequent microscopic examination. Area percentage of immune expression levels was recorded. For tissue section analysis, full HD microscopic imaging system (Leica Microsystems GmbH, Wetzla, Germany) operated by Leica Application software was utilized.

### 2.9. Protein Assessment by ELISA 

Heart tissue was homogenized using PBS, 0.1 M, pH 7.4. Then, bicinchoninic acid (BCA) protein assay kit (Sigma-Aldrich, Saint Louis, MO, USA) was used to determine total protein levels. Measurement of the concentration of phosphorylated protein kinase B (p-PKB) (pAKT) was conducted using a rat ELISA assay kit CSB-E139 11r (CUSABIO, Technology, LLC, Houston, TX, USA). Moreover, phosphatidylinositol 3-kinase (PI3K) were determined using rat ELISA assay kit (FineTest, Wuhan Fine Biotech Co., Ltd., Wuhan, China). In addition, nuclear factor-κB p65 (NF-κB p65) was determined using ELISA assay kit (Elabscience, Biotech, Co., Ltd., Huston, TX, USA). Tumor necrosis factor alpha (TNF-α) and interleukin-6 (IL-6) were assessed by ELISA assay kit (Bioassay, Biotech, CO., Ltd., Hangzhou, China). Microtubule-associated protein 1 light chain 3 beta (MAP1LC3B) (LC3B) was determined by rat ELISA assay kit (LS-F23358 lifespan biosciences, Inc., Seattle, WA, USA). Sequestosome 1 (p62/SQSTM1) was measured by rat ELISA assay DEIA7391 (Creative Diagnostic, Ramsey Road Shirley, NY, USA).

### 2.10. Reverse Transcription-Quantitative Real-Time Polymerase Chain Reaction (RT-qPCR)

Extraction of total RNA was performed using Qiagen tissue extraction Kit (Qiagen, Germantown, MD, USA). Then, cDNA was obtained using high-capacity cDNA Synthesis Kit (Thermo Scientific Co., Waltham, MA, USA). qRT-PCR was carried out using power-up SYBR (Applied Biosystems) utilizing an ABI 7500 RT-PCR System (Applied Biosystems, Foster City, CA, USA). PCR primer pair sequences are shown in Table 1. Relative expression of the studied mitochondrial oxidation genes, including manganese superoxide dismutase (MnSOD) and uncoupling protein-2 (UCP2), was determined. Moreover, mitophagy-related genes, including phosphatase and tensin homolog (PTEN)-induced putative kinase 1 (PINK 1), Parkin RBR E3 ubiquitin protein ligase (Parkin), FUN14 domain-containing 1 (FUNDC1), unc-51-like autophagy activating kinase 1 (ULK1), B-cell lymphoma 2/adenovirus E1B 19 kDa protein-interacting protein 3 (BNIP3) genes, were normalized to the expression level of β-actin gene. The relative quantification was then calculated by the expression 2^−ΔΔCt^.

### 2.11. Statistical Analysis

Data are expressed as mean ± SEM. Parametric data were compared by analysis of variance (ANOVA) using Tukey’s multiple comparison test as post-hoc test. Statistical analyses were implemented using the IBM SPSS statistics (V.19.0, IBM Corp., Armonk, NY, USA, 2010).

## 3. Results

### 3.1. Characterization and Tracing of BM-MSCs-EV 

**Figure 2:**
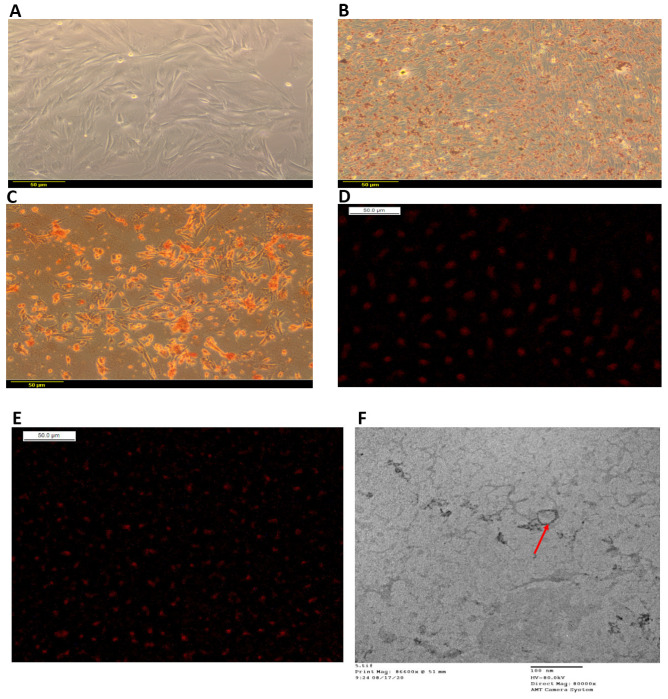
BM-MSCs multipotent differentiation and migration and BM-MSCs-EV vesicular structure. (**A**) BM-MSCs showing a fibroblastic uniform morphological appearance, (**B**) Adipogenic differentiation of BM-MSCs stained with oil red, (**C**) Osteogenic differentiation of BM-MSCs stained with Alizarin red, (**D**) Localization of PKH26-labeled BM-MSCs in the heart of NASH group, (**E**) Localization of PKH26-labeled BM-MSCs in the liver of NASH group, (**F**) TEM of BM-MSCs-EV showing the vesicular structures of BM-MSCs-derived EV.

Isolated BM-MSCs showed a uniform fibroblastic appearance with fibroblastic adhesion properties (Figure 2A). Successful adipogenic differentiation was maintained by BM-MSCs (Figure 2B). Additionally, BM-MSCs showed osteogenic differentiation capacity (Figure 2C). Vesicular structures of BM-MSCs-derived EV were shown by TEM (Figure 2F). Successful homing of BM-MSCs to the heart and liver in the NASH group was achieved as observed by BM-MSCs labeled with PKH26 (Figure 2D,E).

For BM-MSCs characterization, significant expression of CD105 with 62% positive cells, and negative expression of CD45 with 7.68%positive cells and of CD14 with 0.2% positive cells, were detected (Figure S1). Surface markers of EV were detected and showed significant expression of CD63 and CD81 in BM-MSCs-derived EV as compared to BM-MSCs (Supplementary Figure S1). Particle size of EV sample showed peaks at 32 nm at 7.4% intensity and 188.7 nm at 92.6% intensity (Supplementary Figure S1).

### 3.2. BM-MSCs-EV and BM-MSCs Treatment Attenuate Histopathological Alterations of NASH and Its Associated Cardiotoxic Events 

**Figure 3:**
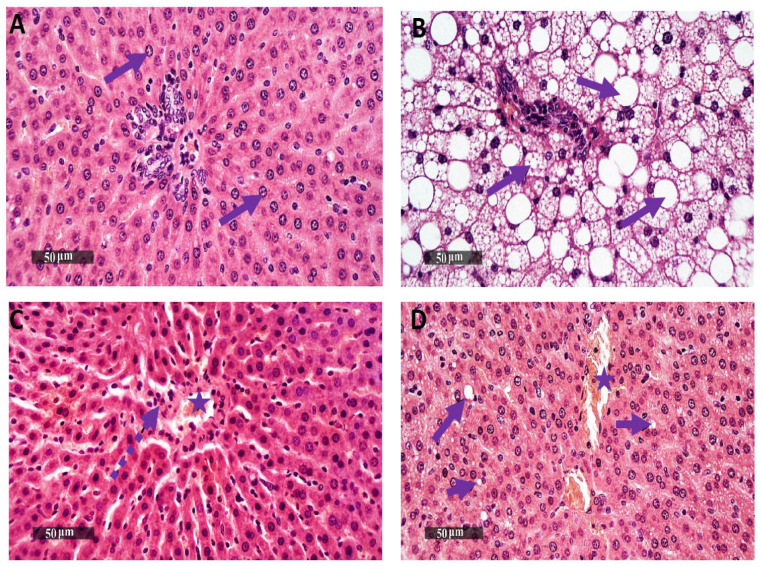

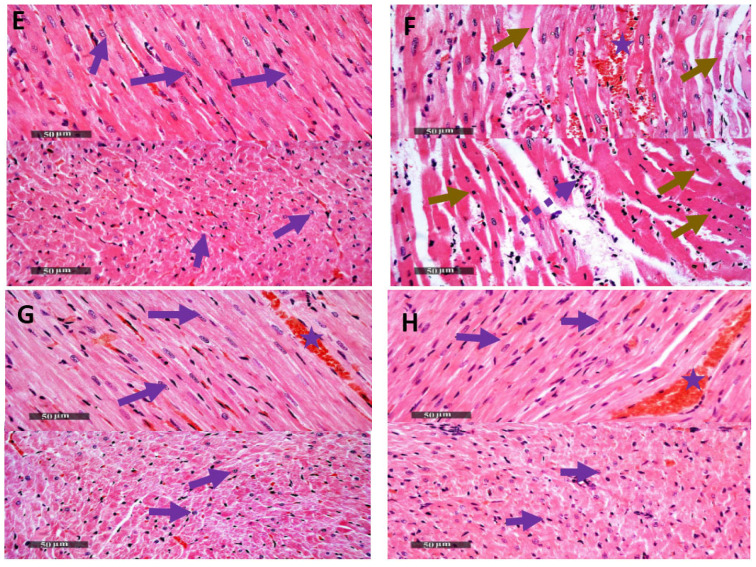
Lane 1: Effects of BM-MSCs-EV and BM-MSCs treatment on histological alteration of the liver tissues (40^x^). Lane 1 shows photomicrographs of liver sections stained by H&E displaying (**A**) Control group with normal histological structures of hepatic parenchyma. All the hepatic lobules show well-organized, intact and radiating hepatocytes with intact subcellular details (arrows). (**B**) NASH group shows severe ballooning, diffuse microvesicular and macrovesicular steatosis (arrows); multiple inflammatory infiltrates are detected. (**C**) BM-MSCs-EV-treated group (120 µg/kg) shows normal hepatic parenchymal cells; some periportal inflammatory cell infiltrates are found (dashed arrow). Congested hepatic blood vessels are detected (star). (**D**) BM-MSCs-treated group depicts mild hepatic steatosis all over hepatic lobules (arrow). Periportal inflammatory cell infiltrates are detected, and congested hepatic blood vessels are shown (star). Lane 2. Effects of BM-MSCs-EV and BM-MSCs treatment on histological alteration of the cardiac tissues (40^x^). Photomicrographs of heart sections stained by H&E Lane 2 showing (**E**) Control group with normal morphological features of cardiac wall, intact endocardium, intact myocardial layer, branched well-organized striated cardiomyocytes, intact subcellular details of cardiomyocytes (arrow). Intact interstitial tissue, intact vasculatures are shown. (**F**) Cardiotoxicity of NASH group shows wide areas of myocardial degenerative and necrotic changes; many figures of nuclear pyknosis and loss of subcellular details are shown (brown arrow); moderate records of fragmented myofibrils, moderate increase in the intercellular spaces are detected. Mild increases in interstitial and perivascular mononuclear cell infiltrates are found (dashed arrow). Moderate focal records of intermuscular hemorrhagic patches are shown (star). (**G**) BM-MSCs-EV (120 µg/kg) -treated group depicts significant protective efficacy with minimal records of myocardial degenerative changes; many apparent intact well-organized cardiomyocytes are shown (arrow); minimal inflammatory cell infiltrates are detected. Persistent milder records of congested intermuscular blood vessels or hemorrhagic patches are observed (star). (**H**) BM-MSCs-treated group shows almost the same protective efficacy as group C samples. Minimal records of myocardial degenerative changes are found. Many apparent intact well-organized cardiomyocytes are observed (arrow). Minimal inflammatory cell infiltrates are detected. Mild records of congested intermuscular blood vessels or hemorrhagic patches are observed (star).

NASH was significantly induced in our model, as histologically examined by stained H&E liver sections, which showed significant ballooning, steatosis and inflammatory infiltrates in the NASH group (Lane 1 Figure 3B). Additionally, NAS was assessed from the histopathological data and showed significant steatosis of score 3, lobular inflammation with score 1 and hepatocellular ballooning of score 2. On the other hand, the control group did not show steatosis, lobular inflammation or hepatocellular ballooning (Lane 1 Figure 3A). Our finding significantly asserts NASH development, as evidenced by significant increase in NAS score in our NASH group by 6-fold compared to control group, which comes in accordance with the histological scoring system for NASH [52]. 

On the contrary, BM-MSCs-EV (120µg/kg) and BM-MSCs significantly reversed hepatic ballooning and steatosis induced by NASH (Lane 1 Figure 3C,D). BM-MSCs-EV (120µg/kg)-treated group showed only lobular inflammation of score 1 with no steatosis or hepatocellular ballooning (NAS score equals 1), while BM-MSCs-treated group showed steatosis of score 1 and lobular inflammation of score 1 with no hepatocellular ballooning (NAS score equals 2).

Histological examination of stained H&E heart sections from different groups reveals normal morphological features of cardiac tissue with intact interstitial tissue accompanied by intact vasculatures in the control group (Lane 2 Figure 3E). On the contrary, cardiotoxicity induced by NASH group showed myocardial degenerative and necrotic changes, as well as nuclear pyknosis with perivascular mononuclear cell infiltrates accompanied by intermuscular hemorrhagic patches (Lane 2 Figure 3F). In contrast, BM-MSCs-EV (120 µg/kg) and BM-MSCs showed significant protective efficacy with minimal myocardial degenerative changes and minimal observed inflammatory cell infiltrates (Lane 2 Figure 3G,H).

### 3.3. BM-MSCs-EV and BM-MSCs Treatment Alleviate Serum Cardiotoxic Markers Associated with NASH

As shown in Table 2, cardiotoxicity has been significantly associated with NASH in our untreated NASH group, with increased serum CK-MB levels, serum LDH levels and serum cTnI levels by 37%, 61% and 103%, respectively, in comparison to the control group. The beneficial effect of BM-MSCs-EV (120 µg/kg) was observed with significant decrease in serum CK-MB, LDH and cTnI levels as compared to cardiotoxicity induced by NASH group. Meanwhile, treatment with BM-MSCs achieved significant decrease in serum CK-MB levels only by 18%, with non-significant decrease in serum levels of LDH and cTnI, as compared to cardiotoxicity induced by NASH group.

### 3.4. BM-MSCs-EV and BM-MSCs Treatment Attenuated Cardiac Hypoxic Responses Induced by NASH

**Figure 4:**
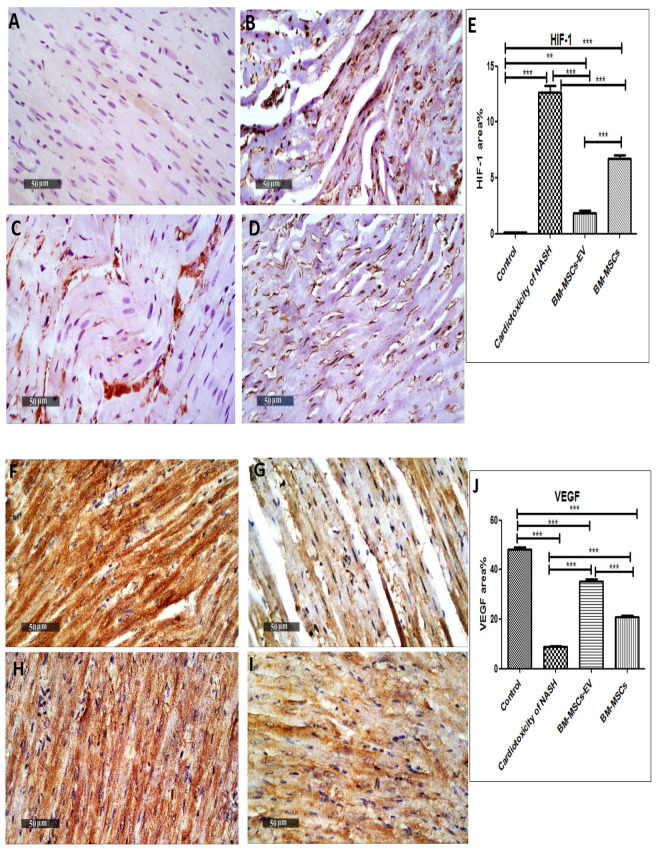
Effects of BM-MSCs-EV and BM-MSCs treatment on the expression of HIF-1 and VEGF in cardiotoxicity of NASH. Immunohistochemical localization of HIF-1 and VEGF in NASH-induced cardiotoxicity. Scale bar, 50 μm. (**A**) Control group, (**B**) Cardiotoxicity of NASH group, (**C**) BM-MSCs-EV (120 µg/kg)-treated group, (**D**) BM-MSCs-treated group. (**E**) Quantitative image analysis of immunohistochemical staining of HIF-1 expressed as area%. (**F**) Control group, (**G**) Cardiotoxicity of NASH group, (**H**) BM-MSCs-EV (120 µg/kg)-treated group, (**I**) BM-MSCs-treated group. (**J**) Quantitative image analysis of immunohistochemical staining of VEGF expressed as area%. For (**E**,**J**), data are presented as mean ± SEM (n = 6). Statistical analysis was carried out by using one-way ANOVA, followed by post-hoc Tukey’s test. ** *p* value < 0.01 and *** *p* value < 0.001.

Cardiac oxygen homeostasis has been significantly disturbed in the NASH group, as demonstrated by significant increase in HIF-1 expression as compared to the control group (Figure 4A,B,E). Treatment with BM-MSCs-EV (120 µg/kg) and BM-MSCs decreased the hypoxic state, as evidenced by a significant decrease in HIF-1 expression by 85% and 47%, respectively, in comparison to cardiotoxicity induced by NASH (Figure 4C–E), with BM-MSCs-EV showing a superior protective effect to BM-MSCs.

### 3.5. BM-MSCs-EV and BM-MSCs Treatment Improved Cardiac Vascular Endothelial Growth Factor in NASH

Functional integrity of cardiac vasculature and angiogenesis has been dramatically affected in the NASH group, with a significant decrease in VEGF expression by 82% when compared to the control group (Figure 4F,G,J). Superior protective effect was observed in BM-MSCs-EV treatment (120 µg/kg), with a significant increase in VEGF expression by 302% as compared to cardiotoxicity induced by NASH. Additionally, BM-MSCs treatment depicted a significant increase in VEGF expression by 136% compared to cardiotoxicity induced by NASH (Figure 4F,I,J). Nevertheless, BM-MSCs-EV-treated group showed a significant increase in VEGF expression by 70% in comparison to BM-MSCs group. 

### 3.6. BM-MSCs-EV and BM-MSCs Treatment Restored Some Cardiac Autophagy Proteins 

**Figure 5:**
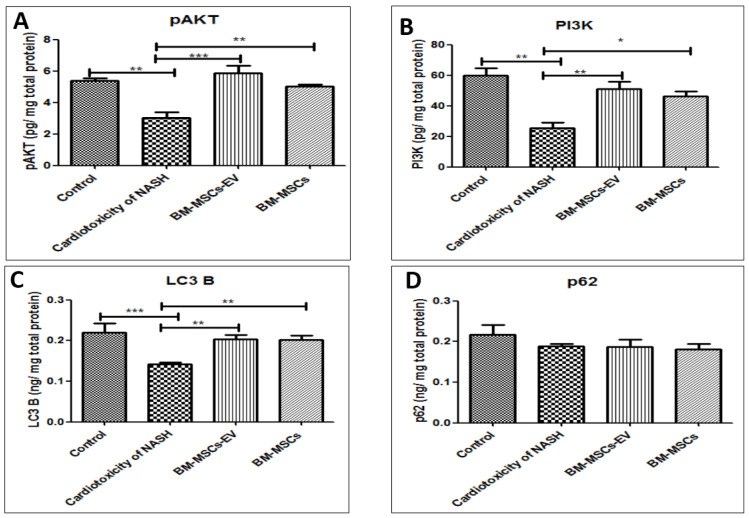
Effects of BM-MSCs-EV and BM-MSCs treatment on cardiac protein expression level of: (**A**) pAKT (**B**) PI3K, (**C**) LC3B, (**D**) p62. Data presented as mean ± SEM (n = 6). Statistical analysis was carried out by using one-way ANOVA, followed by post-hoc Tukey’s test. * *p* value < 0.05, ** *p* value < 0.01 and *** *p* value < 0.001.

Myocardial cell damage and impaired myocardial growth were significantly dysregulated in the NASH group with significant decrease in pAKT and PI3K protein expression by 43% and 58%, respectively, in cardiotoxicity of NASH group when compared to the control group (Figure 5A,B). On the other hand, the activation of pAKT and PI3K pathway was significantly induced by BM-MSCs-EV (120 µg/kg) or BM-MSCs treatment, where BM-MSCs-EV-treated group exhibited significantly increased pAKT and PI3K protein expression by 93% and 101%, respectively, as compared to cardiotoxicity induced by NASH group. In addition, treatment with BM-MSCs significantly increased pAKT and PI3K protein expression by 66% and 82%, respectively, compared to cardiotoxicity induced by NASH group (Figure 5A,B).

Furthermore, myocardial autophagy adaptor LC3B was significantly disturbed in the NASH group, which reflects disrupted myocardial autophagy in the NASH group. This finding was evidenced by significant decrease in LC3B protein expression by 30% as compared to the control group. In contrast, treatment with BM-MSCs-EV (120 µg/kg) or BM-MSCs significantly increased LC3B protein expression by 81% as compared to cardiotoxicity induced by NASH group (Figure 5C). No significant differences were reported in cardiac p62 expression levels among all the studied groups (Figure 5D).

### 3.7. BM-MSCs-EV and BM-MSCs Treatment Improved Some Mitochondrial Antioxidant Markers and Some Mitochondrial Mitophagy Markers 

**Figure 6:**
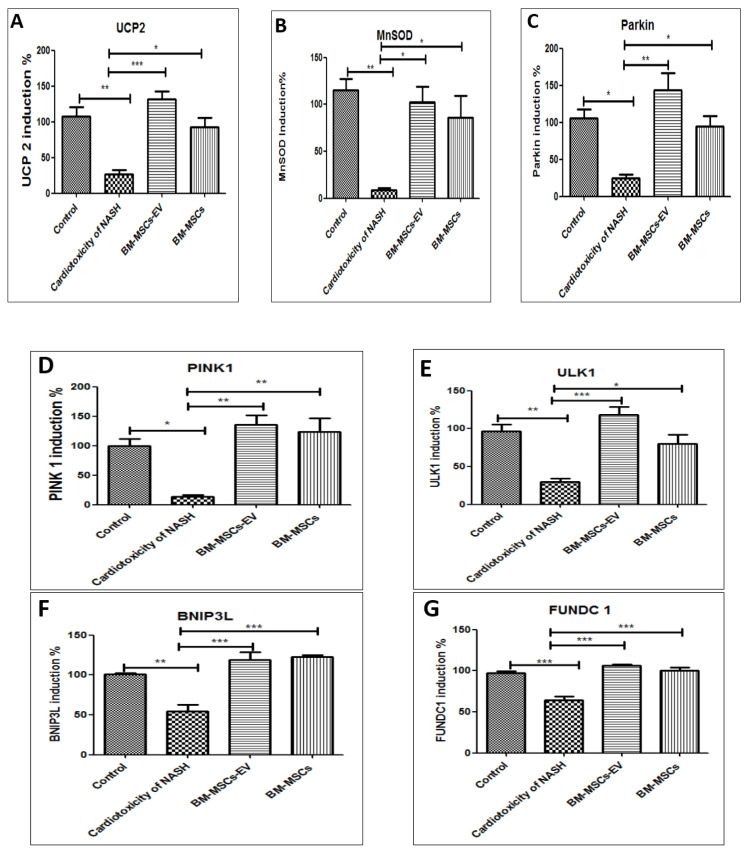
Effects of BM-MSCs-EV and BM-MSCs treatment on cardiac mitochondrial oxidation and mitophagy-related genes in cardiotoxicity of NASH group. (**A**) UCP2 (**B**) MnSOD, (**C**) Parkin, (**D**) PINK1, (**E**) ULK1, (**F**) BNIP3L and (**G**) FUNDC 1. Data presented as mean ± SEM (*n* = 6). Statistical analysis was carried out using one-way ANOVA, followed by post-hoc Tukey’s test. * *p* value < 0.05, ** *p* value < 0.01 and *** *p* value < 0.001.

Malfunctioned mitochondria were significantly associated with NASH group, as shown by the significant decrease in mitochondrial antioxidant gene UCP2 and MnSOD expression by 0.76- and 0.93-fold, respectively, as compared to the control group. Protectively, treatment with BM-MSCs-EV (120 µg/kg) and BM-MSCs significantly increased UCP2 gene expression by 5- and 3.5-fold, respectively, accompanied with significant increase in MnSOD gene expression by 11- and 9-fold, respectively, as compared to cardiotoxicity induced by NASH group (Figure 6A,B).

Interestingly, myocardial Parkin-dependent and -independent mitophagies were significantly deranged in the NASH group. Parkin-dependent mitophagy was significantly downregulated, as evidenced by significant decrease in Parkin and PINK1 gene expressions by 0.77- and 0.86-fold when compared to the control group (Figure 6C,D). Additionally, Parkin-independent mitophagy was also significantly downregulated in NASH-induced cardiotoxicity group, as shown by significant reduction in ULK1, BINP3L and FUNDC 1 gene expressions by 0.7-, 0.47- and 0.35-fold, respectively, when compared to the control group (Figure 6E–G). On the contrary, treatment with BM-MSCs-EV (120 µg/kg) or BM-MSCs reversibly restored Parkin-dependent and -independent mitophagies, as depicted in Figure 6C–G.

### 3.8. BM-MSCs-EV and BM-MSCs Treatment Ameliorated the Cardiac Inflammatory Status Induced by NASH

**Figure 7:**
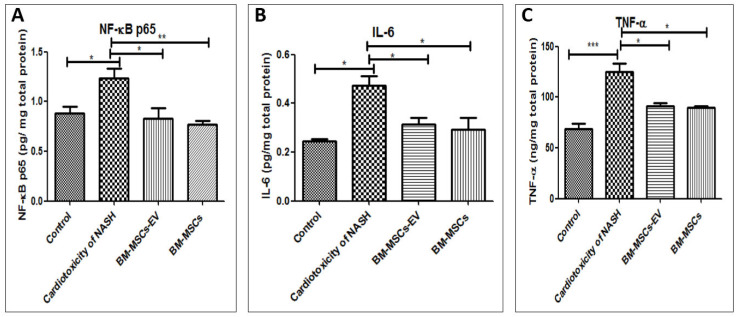
Effects of BM-MSCs-EV and BM-MSCs treatment on cardiac inflammatory markers in cardiotoxicity of NASH group. (**A**) NF-Κb P65 (**B**) IL-6 and (**C**) TNF-α. Data presented as mean ± SEM (n = 6). Statistical analysis was carried out using one-way ANOVA, followed by post-hoc Tukey’s test. * *p* value < 0.05, ** *p* value < 0.01 and *** *p* value < 0.001.

Cardiac inflammation was significantly induced by NASH, as evidenced by significant increase in cardiac levels of NF-κB, IL-6 and TNF-α in the cardiotoxicity group by 39%, 92% and 80.7%, respectively, as compared to the control group (Figure 7). On the contrary, treatment with BM-MSCs-EV (120 µg/kg) significantly decreased the cardiac levels of NF-κB, IL-6 and TNF-α by 33%, 33.7% and 27%, respectively, as compared to cardiotoxicity induced by NASH group (Figure 7). In the same line, treatment with BM-MSCs showed significant decrease in the cardiac levels of NF-κB, IL-6 and TNF-α by 37.6%, 38.3% and 27.9%, respectively, as compared to cardiotoxicity induced by NASH group. 

### 3.9. BM-MSCs Secreted miRNAs and the Signaling Pathways in Cardiotoxic State Induced by NASH 

As shown in Table 3, four key pathways and some targets controlling several cardiac mechanisms were found to be associated with several miRNAs of BM-MSCs according to data extracted from IPA analysis and based on interaction data from the miRPathD. 

The first pathway was PI3K/AKT, which was found to be modulated by let-7a-5p, miR-143-3p, miR-125b-5p, miR-27a-3p, miR-29b-3p, miR-16-5p, miR-34a-5p, miR-199a-3p, miR-26a-5p, miR-30c-5p, miR-145-5p, miR-23a-3p, miR-10a-5p, miR-214-3p, miR-181a-5p, miR-218-5p, miR-196a-5p, miR-100-5p, miR-221-3p, miR-31-5p, miR-103-3p, miR-101-3p. 

Meanwhile, hypoxia signaling was regulated by let-7a-5p, miR-143-3p, miR-125b-5p, miR-27a-3p, miR-29b-3p, miR-16-5p, miR-34a-5p, miR-199a-3p, miR-26a-5p, miR-30c-5p, miR-145-5p, miR-23a-3p, miR-10a-5p, miR-214-3p, miR-221-3p, miR-31-5p.

VEGF signaling was modulated by let-7a-5p, miR-143-3p, miR-125b-5p, miR-27a-3p, miR-29b-3p, miR-16-5p, miR-34a-5p, miR-199a-3p, miR-181a-5p, miR-218-5p, miR-221-3p, miR-31-5p, miR-21-5p.

In addition, NF-κB signaling was found to be regulated by let-7a-5p, miR-143-3p, miR-125b-5p, miR-27a-3p, miR-29b-3p, miR-16-5p, miR-26a-5p, miR-30c-5p, miR-145-5p, miR-23a-3p, miR-10a-5p, miR-214-3p, miR-181a-5p, miR-218-5p, miR-196a-5p, miR-100-5p, miR-21-5p, miR-191-5p, miR-221-3p.

Besides, several targets were regulated by specific miRNAs, whereas MnSOD was found to be regulated by miR-221-3p. In addition, UCP2 was regulated by miR-16-5p. Moreover, VEGFA was found to be regulated by miR-34a-5p and miR-16-5p. However, mitophagy-related target BNIP3L was regulated by miR-221-3p. Inflammatory markers TNF-α and IL-6, were modulated by miR-21-5p. Finally, HIF-1 was also found to be modulated by miR-31-5p and miR-199a-5p.

## 4. Discussion

Mortality threats in NASH have been augmented by the multifaceted systemic complications that extend beyond the liver [53]. Of these, CVDs is one of the leading causes of NASH-associated morbidity and death. This study sought to investigate the molecular mechanisms that control the mitochondrial, hypoxic, angiogenic and inflammatory status in NASH-induced cardiotoxicity. In addition, we aimed to investigate the comparative therapeutic effects of BM-MSCs-derived EV and BM-MSCs in NASH-induced cardiotoxicity, aiming to provide evidence for their reparative potential while avoiding the side effects that might be induced by stem cell therapy [54]. This was achieved through studying the miRNA paradigm of BM-MSCs that controls PI3K/AKT, hypoxic, VEGF and inflammatory signaling pathways in cardiotoxic state induced by NASH. 

The establishment of a NASH model has been successfully achieved by feeding rats with HFD for three months, as agreed with previous studies [19,30]. NASH was confirmed with gross histological ballooning, steatosis and inflammatory infiltrates in the liver. This was accompanied by an increased NAS score, reflecting NASH development as previously documented [30,52]. More interestingly, our results confirmed histological cardiac alterations associated with NASH, as represented by myocardial degeneration and necrotic changes with nuclear pyknosis accompanied by inflammatory infiltrates and intermuscular hemorrhagic patches. This was in agreement with previous studies linking NAFLD with altered myocardial structure and function, as well as premature CV events [1,55]. Moreover, our study confirmed significant increase in serum cardiac toxic markers in the NASH group, which also reflects the development of cardiac toxicity with NASH. 

Accumulating evidence asserted that mitochondrial dysfunction is a crucial factor for NASH development and progression [56], i.e., accumulated hepatic lipids induce accelerated lipid peroxidation, which increases the generation of reactive oxygen species (ROS) and inflammatory cytokines, with subsequent impairment of the respiratory chain, either directly or indirectly, through oxidative damage of the mitochondrial DNA [57]. This subsequently leads to depletion of overall ATP pool and energy status [58]. This in turns affects organs with high energy demand such as the heart, leading to a progressive decline of the mitochondrial function associated with loss of the mitochondrial structural integrity, resulting in cardiomyocytes apoptosis and leading to several cardiac pathologies in NASH [59]. 

Our study showed decreased expressions of cardiac mitochondrial antioxidant markers, such as UCP2, in the NASH group. This finding lies in accordance with previous studies showing the importance of UCP2 in reducing mitochondrial oxidative stress and modulating mitochondrial function [60]. Moreover, our results showed significant downregulation of cardiac MnSOD expression in the NASH group, which asserts our finding of increased oxidative stress state in the heart of NASH group. Besides being an antioxidant enzyme located in the mitochondrial matrix, MnSOD acts as a guardian against the damaging effects of ROS in different cardiac pathologies [61]. 

Protectively, mitophagy can clear up dysfunctional mitochondria [62]. Accordingly, impaired mitophagy in the heart could lead to their accumulation, aggravating the condition and accelerating the progression of diverse CVDs [63]. Hence, therapeutic restoration of disrupted mitophagy can be considered an optimum goal for CVDs and beyond. NASH is considered primarily a mitochondrial disease, with impaired mitophagy being widely studied in NASH livers [1,14]; however, reflecting this impairment on NASH-induced cardiotoxicity is far from clear. 

To our knowledge, this study is the first to show impaired cardiac Parkin-dependent and -independent mitophagies in NASH. This finding was revealed by significant downregulation of Parkin, PINK1, ULK1, BNIP3L and FUNDC1 gene expressions in cardiac tissues of NASH group. In accordance, numerous reports related the disruption of Parkin-dependent and -independent mitophagies with several cardiomyopathies [64], and Parkin depletion was recently shown to contribute to the increased CV risk in the setting of obesity [65]. Additionally, pharmacological upregulation of PINK1/Parkin-mediated mitophagy was found to protect against heart failure [66]. More interestingly, it was found that ULK1 upregulation prevents obesity-induced cardiac dysfunction through regulating lipid metabolism [67]. Besides, deficiency of BNIP3L (also known as NIX) exhibited cardiomegaly and contractile depression, as it plays a major role in the maintenance of mitochondrial homeostasis via mitophagy [68]. Moreover, previous reports showed that deficiency of FUNDC1 leads to mitophagy dysregulation, as it disrupts mitochondrial quality, which in turns aggravates obesity and instigates cardiac dysfunction [69,70]. 

BNIP3 and FUNDC1 are considered LC3B receptors, where they have been extensively characterized in mitophagy-related CV studies [71]. In alignment, our results showed significant reduction in the cardiac expression of the final autophagic marker LC3B in the NASH groups, which plays a key role during autophagosome and mitophagosome formation [72] and thus reflects deficit autophagic/mitophagic mechanisms in cardiac tissues of NASH. Additionally, this finding agrees with previous studies, which showed decreased LC3B gene expression in patients with coronary artery disease, indicating decreased autophagosome formation [73]. However, no significant difference was reported in cardiac expression of p62 in the NASH group as compared to all studied groups. This could possibly be explained by the reported alteration of p62 levels in relation to different stages of cardiac damage and according to different cardiac pathologies [74,75]. 

Mechanistically, our results showed that disrupted mitophagy and mitochondrial antioxidant states in NASH-induced cardiotoxicity might be mediated through PI3K/AKT signaling. This was evidenced by significant decrease in cardiac PI3K and pAKT protein in the NASH group. Additionally, AKT pathway protects cardiomyocytes from hypoxic apoptosis through modulating mitochondrial function, cellular growth and differentiation [76]. This agrees with our finding of increased cardiac expression of HIF-1 in NASH. Being a master regulator of oxygen homeostasis, HIF-1 was found to be increased in myocardial hypoxia [77], which affects the criticality of the mitochondrial function, as it controls oxygen delivery and utilization and redox homeostasis [78]. Moreover, increased cardiac oxygen tension dramatically reduces the expression of VEGF [79], which was previously reported in multiple CVDs with poor clinical outcomes [80]. In the same context, our results showed significant reduction in cardiac level of VEGF in the NASH group. In fact, a double-sided relationship was reported between VEGF and heart physiology, where previous studies proved that VEGF could induce morphogenesis, contractility and thus activate cardiomyocytes. However, others showed that VEGF was progressively induced with inflammation along with cytokine production [81]. Besides, increased levels of VEGF were reported in different CVDs, while others showed a borderline elevation in NASH patients [82]. This could be explained by the wide heterogeneity of CVDs with respect to their multiple etiologies and in relation to the cardiac performance at each stage of the disease [83]. 

Chronic inflammation is one of the hallmarks of various CVDs [84]. The heart is dramatically affected in NASH owing to its high mitochondrial metabolic activities. In agreement, our study showed an activated cardiac inflammatory state evidenced by increased levels of activated cardiac NF-κB p65, cardiac IL-6 and cardiac TNF-α in NASH-induced cardiotoxic group, which all assures an increased inflammatory state in cardiac tissues of NASH, in alignment with previous studies [85,86]. 

Our second aim was to compare the therapeutic potential of BM-MSCs-derived EV with BM-MSCs in NASH-induced cardiotoxicity, scrutinizing the effect of the secreted miRNAs paradigm, which regulates the molecular mechanisms that control multiple mechanisms in cardiac tissue. Previously, multiple works pointed to the validity of BM-MSCs as a successful therapeutic option in a vast array of CVDs [87,88,89]. However, BM-MSCs have not yet been investigated in cardiotoxic events induced by NASH. 

We herein showed perfect homing of labeled BM-MSCs to the liver and heart. Importantly, our results highlighted a superior protective effect of BM-MSCs-derived EV to BM-MSCs in decreasing serum cardiotoxic markers, restoring cardiac histological alterations, decreasing hypoxic state and increasing vascular growth factors. This is in line with previous studies suggesting alternatives to stem cell therapy with the same reparative potential but with reduced side effects [90,91]. 

Beneficially, both BM-MSCs-derived EV and BM-MSCs were found to restore mitochondrial antioxidant state in cardiac tissues of NASH. This was explored with significant increase in cardiac expression of mitochondrial antioxidants UCP2 and MnSOD. These findings were in accordance with previous reports, which showed potential antioxidant paradigm of mesenchymal stem cell therapy [92]. In the same line, recent studies highlighted that EV derived from BM-MSCs could regulate oxidative damage in cardiac tissue [93]. 

More interestingly, BM-MSCs-derived EV and BM-MSCs were also proved to activate disrupted cardiac mitophagy in NASH. In this regard, significant upregulation of Parkin, PINK1, ULK1, BNIP3L and FUNDC1 gene expressions was observed accompanied by significant cardiac increase in LC3B, pAKT and PI3K protein expression in NASH. In alignment, recent studies confirm the therapeutic potential of MSCs and their derived EV against myocardial damage, which might be mediated through activating cardiac autophagy [94,95]. 

In addition, BM-MSCs-derived EV and BM-MSCs were able to inhibit cardiac inflammation, hypoxia and induce vascular angiogenesis, as evidenced by restoring HIF-1, NF-κB, IL-6 and TNF-α levels and increasing cardiac VEGF levels. These findings are in accordance with the previously reported anti-inflammatory, anti-hypoxic and pro-angiogenic effects of BM-MSCs-derived EV and BM-MSCs in different diseases [96,97].

Our study introduced a paradigm of complex array of miRNAs found in BM-MSCs, which were found to collectively regulate interrelated and integrated signaling pathways, such as PI3K/AKT, hypoxia, VEGF and inflammatory signaling, to induce possible protective effects on the cardiac tissues. Based on the important roles played by miRNAs in regulating the expression of several genes, miRNA-based therapeutic potential of stem cells is introduced as a vital therapeutic option for multiple diseases [98]. Importantly, our results lie in accordance with previous studies, which showed some common miRNAs derived from exosomes or EVs derived from mesenchymal stem cells regulate different functions in various diseases [29,99,100,101]. 

To our knowledge this is the first study that showed a comprehensive possible interaction between miRNAs derived from BM-MSCs and their regulated molecular signaling pathways and targets in the cardiotoxic state induced by NASH, which opens the door for further research. 

Although standardized methods for EV quantification remain to be established, this study has some limitations where it depends on protein quantifications only for EV dosing. However, particle counts, total lipid quantification, quantification of total RNA and detecting specific molecules are also reported for EV quantification for dosing [102]. Moreover, our study depends on the IV route and on multiple dosing in EV application; however, different routes of administration and multiple dosing are also reported for different EV therapeutics [102]. These discrepancies in EV quantification, dosing and administration highlight the limitation associated with current therapeutic of EV. However, it paves the road for future studies to standardize this promising field. 

## 5. Conclusions

Complex modulation of mitochondrial functions, hypoxia, angiogenesis and inflammatory pathways are crucial in controlling the cardiotoxic events induced by NASH. We herein showed that BM-MSCs-derived EV and BM-MSCs restored cardiac mitochondrial antioxidant states, as well as Parkin-dependent and -independent mitophagies, through PI3K and pAKT pathways. Besides, they reduced cardiac hypoxia, inflammation and increased cardiac angiogenesis. These effects were suggested to result from a paradigm of secreted miRNAs that were found to modulate PI3K/pAKT, hypoxia, VEGF and NF-Κb signaling pathways and regulate several targets in the heart. Our findings unravel the potential ameliorative effects of BM-MSCs-EV as a comparable new avenue for BM-MSCs for modulating NASH-induced cardiotoxicity. Future clinical studies are required to confirm our findings. 

## Figures and Tables

**Table 1:** Sequences of primer sets used for gene expression analysis.

Gene Symbol	Primer Sequence	GenBank Accession Number
MnSOD F: MnSOD R:	5′-GTGCAGGTAAGTGGCAGGG-3′ 5′-TCGTGGTACTTCTCCTCGGT-3′	NM_017051.2
UCP2 F: UCP2 R:	5′-CGTCTGCACTCCTGTGTTCT-3′ 5′-TGTTGAGTGGGGCATTGTGT-3′	NM_019354.3
PINK1 F: PINK1 R:	5′-GACCTCAGCCTGCTCTTCTG-3′ 5′-GCTTTGCTGGGACACCTTTG-3′	NM_001106694.1
Parkin F: Parkin R:	5′-CCAGGTACAATCTCTCCGCG-3′ 5′-AAGTTGCGATCTCCACCAGG-3′	NM_020093.1
FUNDC 1 F: FUNDC 1 R:	5’-ACATTGTGATATCCAGCGGC-3’ 5’-AAGCTTCAGGTGGCAAAGTG-3’	NM_001025027.1
ULK1 F: ULK1 R:	5’-GTTGCTGACTCCAAGCCAAA-3’ 5’-ATCTTGGAGGACGAAAGCCA-3’	NM_001108341.1
BNIP3L F: BNIP3L R:	5’-TGCCCTATCACAAAGCAGGA-3’ 5’-GGTCCAACAAACGCTTCACT-3’	NM_080888.2
β-actin F: β-actin R:	5′-TGTCACCAACTGGGACGATA-3′ 5′-GGGGTGTTGAAGGTCTCAAA-3′	NM_031144.3

MnSOD, manganese superoxide dismutase; UCP2, uncoupling protein-2; PINK 1, phosphatase and tensin homolog (PTEN)-induced putative kinase 1; Parkin, Parkin RBR E3 ubiquitin protein ligase; ULK1, unc-51-like autophagy activating kinase 1; FUNDC1, FUN14 domain-containing 1; BNIP3, B-cell lymphoma 2/adenovirus E1B 19 kDa protein-interacting protein 3; β-actin, beta actin.

**Table 2:** Effect of BM-MSCs-EV and BM-MSCs treatment on serum cardiotoxic markers.

Groups	CK-MB (U/L)	LDH (U/L)	cTnI (ng/mL)
Control	29.2 ± 0.4	39.7 ± 4.4	1.42 ± 0.2
Cardiotoxicity of NASH	40 ± 0.6 ^a^	64 ± 5.7 ^a^	2.89 ± 0.09 ^a^
BM-MSC-EV	31.1 ± 0.38 ^b^	39.5 ± 3.08 ^b^	1.72 ± 0.3 ^b^
BM-MSCs	32.7 ± 1.9 ^b^	47.8 ± 7.1	1.79 ± 0.3

Data presented are means ± SEM (*n* = 10). Statistical analysis was performed by one-way ANOVA, followed by Tukey’s post-hoc test. ^a^ Significant difference as compared to the control group. ^b^ Significant difference as compared to the NASH-induced cardiotoxicity group.

**Table 3:** List of identified miRNAs and their associated signaling pathways and targets.

miRNAs	Signaling Pathways and Targets
let-7a-5p, miR-143-3p, miR-125b-5p, miR-27a-3p, miR-29b-3p, miR-16-5p	PI3K/AKT, NF-κB, VEGF Hypoxia
miR-34a-5p, miR-199a-3p	PI3K/AKT, VEGF Hypoxia
miR-26a-5p, miR-30c-5p, miR-145-5p, miR-23a-3p, miR-10a-5p, miR-214-3p	PI3K/AKT, NF-κB, Hypoxia
miR-181a-5p, miR-218-5p	PI3K/AKT, NF-κB, VEGF
miR-196a-5p, miR-100-5p	PI3K/AKT, NF-κB
miR-221-3p, miR-31-5p	PI3K/AKT, VEGF Hypoxia
miR-21-5p	NF-κB, VEGF
miR-103-3p, miR-101-3p	PI3K/AKT
miR-221-3p	MnSOD
miR-16-5p	UCP2
miR-34a-5p, miR-16-5p	VEGFA
miR-221-3p	BNIP3L
miR-31-5p, miR-199a-5p	HIF-1
miR-21-5p	TNF-α, IL-6
miR-191-5p, miR-221-3p	NF-κB

## Data Availability

All data are provided within the manuscript.

## Supplementary Materials

The following supporting information can be downloaded at: https://www.mdpi.com/article/10.3390/life12030355/s1, Figure S1. (**A**) Flow cytometry curve showing a positive control, (**B**) Flow cytometry curve showing the expression of CD105 in BM-MSCs, (**C**) Flow cytometry curve showing the expression of CD45 in BM-MSCs, (**D**) Flow cytometry curve showing the expression of CD14 in BM-MSCs, (**E**) Flow cytometry curve showing the expression of CD63 in BM-MSCs-EV, (**F**) Western blot for surface markers (CD81, CD63, GAPDH), (**G**) Particle size for BM-MSCs-EV sample.
