# Supplementary figures and images for: Bone Marrow Mesenchymal Stem Cells and Their Derived Extracellular Vesicles Attenuate Non-Alcoholic Steatohepatitis-Induced Cardiotoxicity via Modulating Cardiac Mechanisms
Source: Life (Basel). 2022 Feb 28;12(3):355. doi: 10.3390/life12030355 (PMC8952775; doi:10.3390/life12030355)

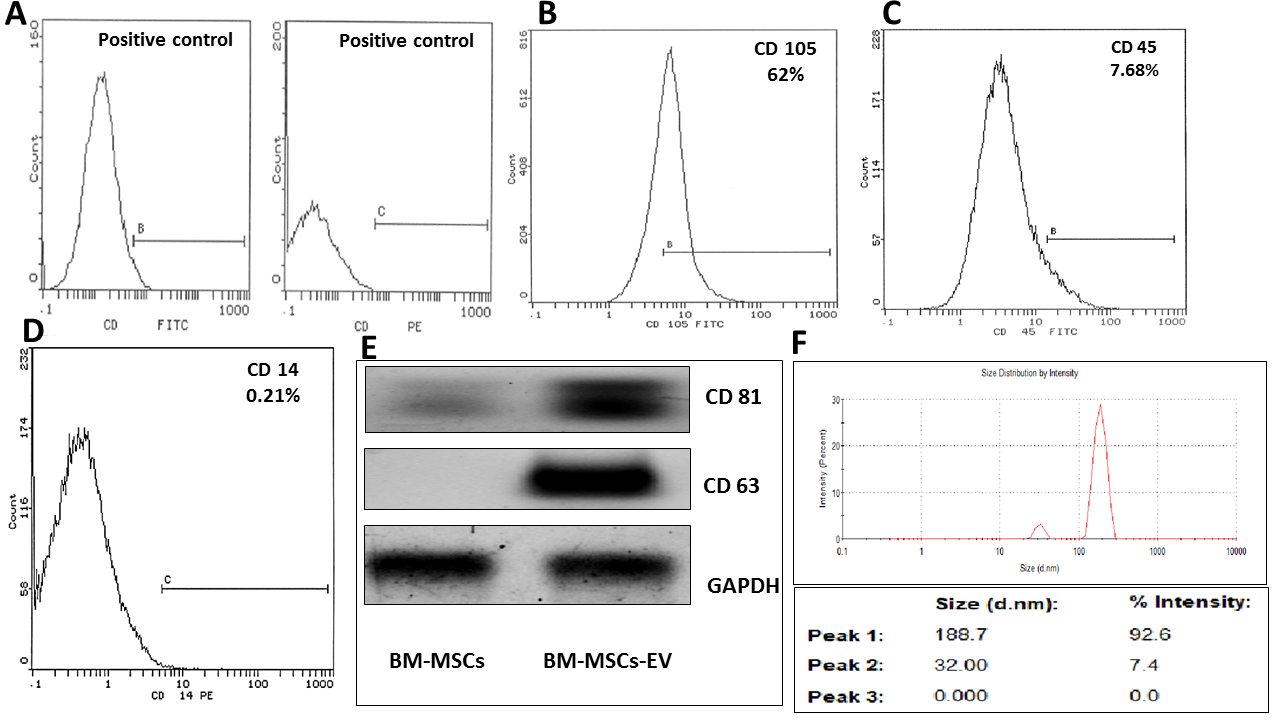

Supplement: Supplementary file 1 [file life-12-00355-s001.zip › life-1570088-supplementary.png]
